# Extensive Deep Vein Thrombosis With Intraabdominal Extension Triggered by Oral Contraceptive Use in a Young Patient With Interrupted Inferior Vena Cava With Azygos Continuation: A Case Report

**DOI:** 10.7759/cureus.42690

**Published:** 2023-07-30

**Authors:** Mosa R Abu Sabha, Zinah A Bairmani, Khadeejeh M Alfroukh, Osayd Nassar, Mutaz Albakri

**Affiliations:** 1 Internal Medicine, Faculty of Medicine, Al Quds University, Jerusalem, PSE; 2 Department of Pharmacology & Experimental Therapeutics, Thomas Jefferson University, Philadelphia, USA; 3 Internal Medicine Department, Al-Ahli Hospital, Hebron, PSE; 4 Internal Medicine Department, Hebron University College of Medicine, Hebron, PSE

**Keywords:** anticoagulation, vena cava variations, azygos continuation, deep vein thrombosis, inferior vena cava anomalies

## Abstract

Congenital malformations of the inferior vena cava (IVC) are rare and often asymptomatic, typically discovered incidentally during imaging. However, these anomalies can result in circulatory stasis, impede venous return, and serve as predisposing factors for thrombus formation. Here, we present a unique case of a 28-year-old female patient who was found to have interrupted IVC with azygos continuation, an exceedingly rare IVC anomaly, during a work-up of extensive bilateral deep vein thrombosis (DVT) with an intraabdominal extension which was triggered by recent combined oral contraceptive pills (OCP) use. This case highlights the importance of considering vena cava malformations as an underlying cause for extensive DVT, even in the absence of conventional risk factors. Clinicians should be aware of the potential association between congenital vena cava anomalies and thrombotic events, as early recognition and appropriate management are crucial for preventing complications such as pulmonary embolism.

## Introduction

During embryogenesis, the formation of the inferior vena cava (IVC) involves a complex process of development, regression, and anastomosis of three sets of paired veins: the posterior cardinal, subcardinal, and supracardinal veins [1]. The normal IVC transforms into a unilateral right-sided system, consisting of distinct segments such as postrenal, renal, prerenal, and hepatic. However, disruptions in the fusion of these originally paired structures can result in various anomalies of the IVC. The estimated prevalence of these anomalies in the general population ranges from 0.07% to 8.7% [2].

One specific anomaly is interrupted IVC with azygos continuation, a rare congenital malformation resulting from abnormal development of the IVC segment during embryogenesis [3]. In this anomaly, the IVC terminates below the hepatic vein, and systemic venous flow beyond that point is accommodated by a dilated azygos vein [4]. Eventually, the blood from the azygos vein empties into the superior vena cava (SVC) via a dilated azygos arch.

Interrupted IVC with azygos continuation has been associated with an increased risk of recurrent deep vein thrombosis (DVT) in the lower limbs [5]. The altered venous hemodynamic and flow patterns caused by this anomaly can lead to circulatory stasis, impairing venous return, and promoting thrombus formation [6]. However, due to the rarity of this condition, limited information is available regarding its precise prevalence and the underlying mechanisms contributing to DVT risk.

In this paper, we present a unique clinical scenario involving a 28-year-old female patient with symptoms of bilateral DVT. Doppler ultrasound confirmed the presence of DVT, and computed tomography (CT) of the abdomen and pelvis with intravenous contrast was performed to assess for intraabdominal extension, which revealed an interrupted IVC, with the inferior vena cava terminating as the right renal vein, and both common iliac veins joining each other and draining into the azygos vein, consistent with azygos continuation of the IVC.

## Case presentation

A 28-year-old female patient presented to the emergency department with a one-day history of bilateral lower limb swelling and pain, which had progressed gradually. The pain was so severe that she could not bear weight. A review of systems was positive for mild left flank pain. She had undergone rhinoplasty two months earlier. One month prior presentation she was diagnosed with ovarian cyst for which she received oral contraceptive pills (OCP) for 21 days. The patient had no history of trauma or relevant family history. The patient was single and without sexual relationships. She smoked Shisha occasionally. She had no history of recent travel.

On clinical examination, the patient was hemodynamically stable and afebrile. Lower extremities were swollen bilaterally and tender on palpation from the foot extending up to the thigh. There were no signs of consolidation, pleural effusion, or pulmonary hypertension. The abdomen was soft and lax without palpable masses or hepatosplenomegaly. The remaining physical examination was unremarkable. 

**Figure 1:**
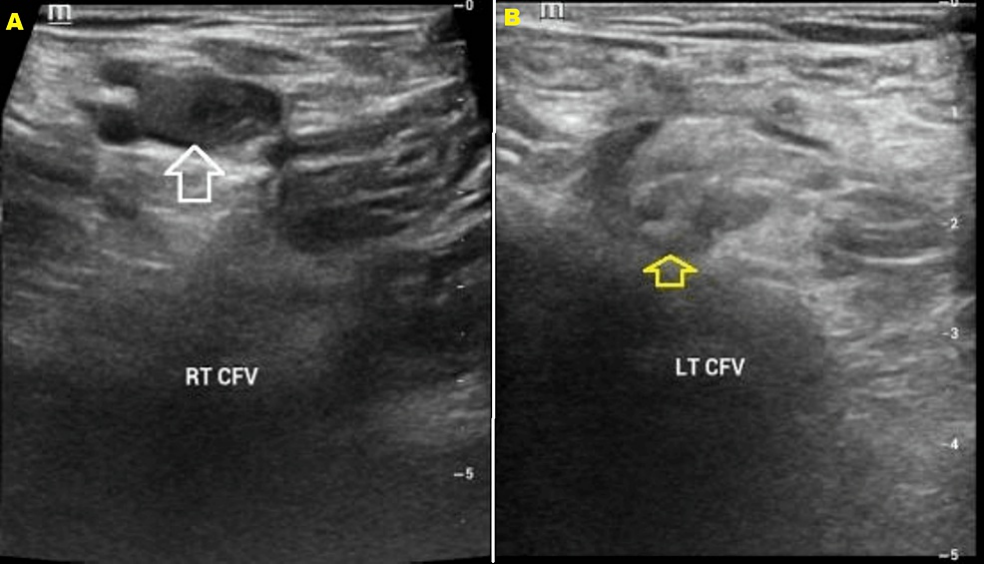
Selected ultrasound images at the level of the right common femoral (A) and left common femoral (B) veins which show a non-compressible vein with an internal filling defect (white and yellow arrows).

A bilateral lower limb venous compression ultrasonography with Doppler was performed which showed an extensive deep vein thrombosis of both lower limbs involving the deep calf veins, popliteal veins, superficial and common femoral veins (Figures 1A, 1B), external iliac veins up to the common iliac veins, and visualized part of IVC, which were dilated and filled with iso-echoic material with no flow seen on color or power Doppler. 

The patient was admitted to the medical ward for comprehensive assessment and treatment. The Initial laboratory investigations and coagulation screen were normal except for elevated C-reactive protein level and D-dimer assay (Table 1). Therapeutic dose of low molecular weight heparin and enoxaparin 60 mg twice daily as subcutaneous injections were initiated.

**Table 1:** Initial laboratory tests on admission.

Lab Test	Result	Reference Range
Hemoglobin	12.3	13.5-17.5 g/dl
White Blood Cells	8000	4500-11000/mm^3^
Platelets	185000	150000-400000/mm^3^
C-reactive protein	148	0-6 mg/L
Prothrombin Time	12.2	11-15 seconds
Activated Partial Prothrombin Time	28	25-40 seconds
D-dimer	29500	0–250 ng/mL

**Figure 2:**
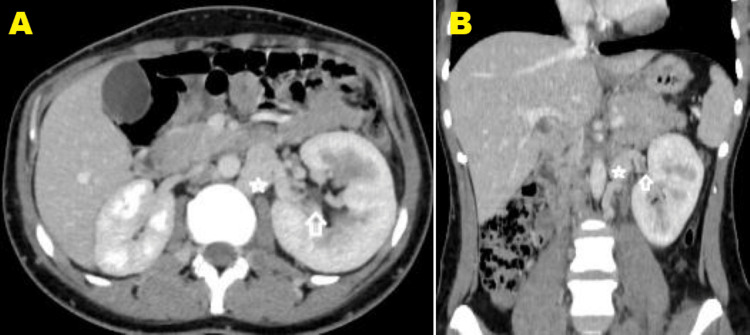
Selected axial (A) and coronal (B) cuts at the level of the left kidney in the venous phase show a filling defect (white arrow) in the left renal vein that drains into the hemizygous vein (white star).

**Figure 3:**
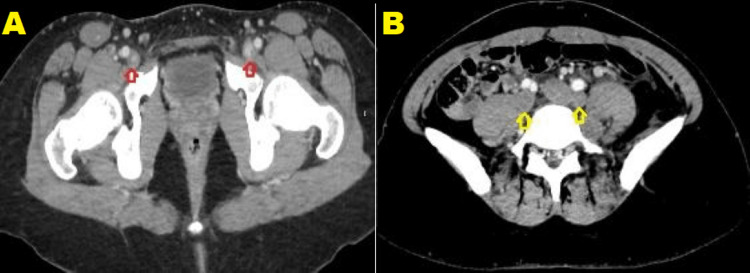
Multilevel axial cuts at the levels of lower pelvis (A) and lower abdomen (B) demonstrating a filling defect in the common iliac veins (red arrow) and the common iliac veins (yellow arrow) as the common iliac veins are draining into the azygos vein.

CT scan of the abdomen and pelvis with IV contrast was performed to look for an intraabdominal extension of the thrombus or other potential abdominal pathology. It revealed that the inferior vena cava is terminated as the right renal vein (Figures 2A, 2B), and both common iliac veins join each other and drain into the azygos vein. These findings are consistent with azygos continuation of inferior vena cava (Figures 3A, 3B). Both common femoral veins, both external and internal iliac and both common iliac veins, and the conjoint vein of both common iliac veins are dilated and showed no enhancement after administration of IV contrast, consistent with extensive proximal DVT (Figures 3A, 3B). The left kidney is drained by two veins: the first one terminates in the hemizygous vein and shows focal dilatation up to 2 cm in a segment measuring 2.6 cm long, left renal vein aneurysm (Figures 2A, 2B). The second renal vein is drained into the left common iliac vein and shows a filling defect along most of its course and is associated with the enlarged left kidney- thrombosis. Few segmental renal veins show filling defect- venous thrombosis. Partially thrombosed left ovarian vein, however, the left ovary is normal in size. Multiple collateral veins were noted.

**Figure 4:**
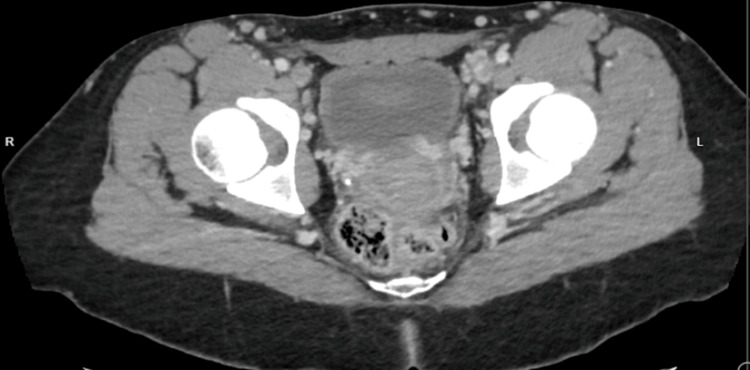
Follow-up axial computed tomography (CT) scan cut at the level of the pelvis demonstrates a filling defect at both external iliac veins bilaterally.

Following the discussion with the vascular surgery team, no interventional procedures including catheter-guided thrombolysis were considered necessary, and it was recommended to continue anticoagulation therapy. She was discharged home in stable condition with improvement in her symptoms after seven days to continue rivaroxaban 15 mg twice daily for 21 days, then 20 mg once daily indefinitely. In the follow-up clinic, the patient was feeling well and active with improvement in the lower limb swelling. A follow-up abdomen and pelvis CT scan conducted three months later revealed the presence of chronic DVT in the common iliac, external iliac (Figure 4), and internal iliac veins. Furthermore, chronic sub-hepatic IVC and left renal vein thrombosis were also identified. Additionally, the scan indicated a small right kidney accompanied by a compensatory hypertrophied left kidney. Notably, a thick-walled cystic lesion was observed in the left adnexal region, potentially originating from the left ovary, with mild pelvic free fluid surrounding it, suggesting a ruptured cyst or a prominent follicle. This finding underscores the significance of IVC anomaly as a crucial risk factor for extensive thrombosis and emphasizes the importance of lifelong anticoagulant therapy to prevent further complications. 

## Discussion

We describe a case of a 28-year-old female patient who presented with extensive bilateral lower extremities and intraabdominal DVT which was provoked by the use of combined OCP in the background of interrupted IVC with azygos continuation. The inferior vena cava is the largest vein in the body, draining blood from the lower extremities, pelvis, and abdominal cavity. It is formed by the union of the left and right common iliac veins at the fifth lumbar vertebra level. The IVC ascends retroperitoneally to the right side of the aorta and vertebral column behind the duodenum, portal vein, and liver and enters the thoracic cavity by piercing in the central tendon of the diaphragm at the eighth thoracic vertebra level. Along its course, the IVC receives many tributaries including paired third and fourth lumbar veins, the right gonadal veins, paired renal veins, the right suprarenal vein, paired inferior phrenic veins, and three hepatic veins. Finally, the IVC is connected with the SVC by the azygous vein [7,8]. 

The development of IVC starts in the fourth week of gestation process of fusion and regression of posterior cardinal, sub-cardinal, supra-cardinal, and vitelline veins. By the end of the eighth week of gestation, the fully-developed IVC will be composed of four segments: hepatic segments (right vitelline vein), suprarenal segment (right sub-cardinal), renal (right supra-cardinal) and infrarenal (right supra-cardinal). The azygous vein is derived from the supra-cardinal [9,10]. Agenesis or failed fusion of these segments can lead to interruption of the IVC [11].

The abnormal persistence or regression of embryological veins results in IVC anomalies [12]. The most common anomalies include duplicated IVC, left-sided IVC, and interruption of the IVC (Table 2).

**Table 2:** The most common inferior vena cava (IVC) anomalies.

Type of Anomaly	Prevalence	Pathophysiology	Comments
Duplicated IVC	0.2%–3% [13]	Abnormal persisting left supra-cardinal vein, resulting in duplicated infrarenal IVC segments.	Can resemble adenopathy or lymph nodes. If not recognized, recurrent pulmonary embolism can occur despite routine infrarenal IVC filter placement [14].
Left-sided IVC	0.2%–0.5% [13]	Abnormal regression of the right supra-cardinal vein and persistence of the left supra-cardinally vein results in a left-sided IVC.	The procedures such as abdominal aortic aneurysm repair, left-sided nephrectomy, and oblique lumbar fusion can be complicated.
Absent infrarenal IVC	unknown	Disruption of the infrarenal IVC or infrarenal agenesis of IVC with azygos continuation.	It is caused by acquired intrauterine or perinatal venous thrombosis, rather than failure of embryonic vein development [12].
Interrupted IVC with azygos continuation	0.6%	Failure of anastomosis between the right subcardinal vein and the vitelline vein.	Classically associated with polysplenia, cardiovascular malformations, and situs anomalies [15].

Despite most of these anomalies being discovered incidentally, they can cause extensive venous thromboembolism, lower extremities venous insufficiency, and pelvic congestion. The risk of DVT is higher if the anomaly is associated with poor collateral [13].

This case report presents a unique clinical scenario involving a young female patient with interrupted IVC with azygous continuation and extensive bilateral deep vein thrombosis of the lower extremities. Although the patient had only one identifiable risk factor for thromboembolism, the use of oral contraceptive pills and the presence of vena cava malformation likely contributed to the extensive bilateral DVT. Moreover, our patient did not have the classic association of heterotaxy polysplenia syndrome, which is a rare group of congenital abnormal arrangements of the abdominal and thoracic organs that are associated with the presence of two or more spleens [16].

The treatment of DVT in the setting of interrupted IVC with azygous continuation focuses on preventing the propagation of thrombus and pulmonary embolism by using systemic anticoagulation especially unfractionated heparin in the acute setting [17]. Given the lifelong risk of venous thrombosis and its complications, the patient should receive anticoagulation therapy with warfarin or direct oral anticoagulant indefinitely. Surgical intervention for thrombectomy, vascular reconstruction, and bypass surgery was indicated in certain cases such as non-healing stasis ulcers, bleeding from a venous aneurysm, and severe acute thrombosis with hemodynamic instability [18].

Our case report highlights the substantial association between IVC anomaly and the risk of DVT. The presence of an IVC anomaly should not be overlooked, as it represents a significant risk factor for extensive thrombosis. It is crucial for healthcare professionals to recognize this anomaly and consider it when assessing patients with DVT or those at elevated risk for thromboembolic events. Moreover, it emphasizes the critical role of indefinite anticoagulant therapy in managing individuals with IVC anomalies. Anticoagulation is vital in preventing the progression of thrombosis and reducing the likelihood of complications such as pulmonary embolism. Clinicians should ensure that appropriate anticoagulant regimens are prescribed and closely monitored to mitigate the risks associated with IVC anomaly-related thrombosis. 

## Conclusions

This case report emphasizes the significance of identifying and understanding congenital anomalies of the IVC in patients presenting with DVT, particularly when conventional risk factors may be present alongside. By increasing awareness of the association between interrupted IVC with azygos continuation and DVT, clinicians can provide appropriate management and potentially prevent future thrombotic events.
